# *HER2–CDH1* Interaction via Wnt/B-Catenin Is Associated with Patients’ Survival in HER2-Positive Metastatic Gastric Adenocarcinoma

**DOI:** 10.3390/cancers14051266

**Published:** 2022-02-28

**Authors:** Valli De Re, Lara Alessandrini, Giulia Brisotto, Laura Caggiari, Mariangela De Zorzi, Mariateresa Casarotto, Gianmaria Miolo, Fabio Puglisi, Silvio Ken Garattini, Sara Lonardi, Renato Cannizzaro, Vincenzo Canzonieri, Matteo Fassan, Agostino Steffan

**Affiliations:** 1 Immunopathology and Cancer Biomarkers, Centro di Riferimento Oncologico di Aviano (CRO) IRCCS, 33081 Aviano, Italy; gbrisotto@cro.it (G.B.); lcaggiari@cro.it (L.C.); mdezorzi@cro.it (M.D.Z.); mtcasarotto@cro.it (M.C.); asteffan@cro.it (A.S.); 2 Surgical Pathology and Cytopathology Unit, Department of Medicine (DIMED), University of Padova, 35128 Padova, Italy; lara.alessandrini@aopd.veneto.it (L.A.); matteo.fassan@unipd.it (M.F.); 3 Unit of Medical Oncology and Cancer Prevention, Department of Medical Oncology, Centro di Riferimento Oncologico di Aviano (CRO) IRCCS, 33081 Aviano, Italy; gmiolo@cro.it (G.M.); fabio.puglisi@cro.it (F.P.); 4 Department of Medicine (DAME), University of Udine, 33100 Udine, Italy; 5 Department of Oncology, University Hospital, 33100 Udine, Italy; silvioken@hotmail.it; 6 Oncology Unit 3, Veneto Institute of Oncology, Istituto Di Ricovero E Cura a Carattere Scientifico (IRCCS), 35128 Padova, Italy; sara.lonardi@iov.veneto.it; 7 Oncological Gastroenterology, Centro di Riferimento Oncologico di Aviano (CRO) IRCCS, 33081 Aviano, Italy; rcannizzaro@cro.it; 8 Pathology Unit, Centro di Riferimento Oncologico di Aviano (CRO) IRCCS, 33081 Aviano, Italy; vcanzonieri@cro.it; 9 Department of Medical, Surgical and Health Sciences, University of Trieste, 34127 Trieste, Italy

**Keywords:** *HER2*, *CDH1*, E-cadherin, gastric cancer, WNT/β-catenin pathway, survival, metastasis, *EGF*, *RARA*, *RPL19*

## Abstract

**Simple Summary:**

A deeper understanding of the molecular mechanisms involved in gastric cacner (GC) pathologenesis would help the identification of prognostic biomarkers and the development of new treatments. Human epidermal growth factor receptor 2 (HER2/ErbB2), a membrane-bound receptor of the EGFR family, may be overexpressed in GC. Trastuzumab is a *HER2* inhibitor used to treat HER2+ metastatic gastric cancer (mGC). The present study aims to investigate the relationship between *CDH1* mRNA expression and HER2-positivity in mGC using a multiplexed gene expression profile in two series of GC patients: 38 HER2+ and HER2- mGC and 36 HER2- GC with and without metastasis. Our results revealed the relationship between *CDH1* and *HER2* mRNA expression in mGC via the canonical WNT/β-catenin pathway and identified *EGF* as an independent prognostic biomarker for survival.

**Abstract:**

Trastuzumab is a human epidermal growth factor receptor 2 (HER2) inhibitor used to treat HER2+ metastatic gastric cancer (mGC). The present study aims to investigate the relationship between *CDH1* mRNA expression and HER2-positivity in mGC using a multiplexed gene expression profile in two series of gastric cancer (GC): Series 1 (*n* = 38): HER2+ and HER2- mGC; Series 2 (*n* = 36) HER2- GC with and without metastasis. To confirm the results, the same expression profiles were analyzed in 354 GC from The Cancer Genome Atlas (TCGA) dataset. The difference in gene expression connected *HER2* overexpression with canonical wingless-type (Wnt)/β-catenin pathway and immunohistochemical (IHC) expression loss of E-cadherin (E-CAD). *CDH1* mRNA expression was simultaneously associated with the rs16260-A variant and an increase in E-CAD expression. Differences in retinoic acid receptor alfa (*RARA*), *RPL19* (coding for the 60S ribosomal L19 protein), catenin delta 1 (*CTNND1*), and epidermal growth factor (*EGF*) mRNA levels—all included in the Wnt/β-catenin pathway—were found associated with overall survival (OS). *RARA*, *CTNND1*, and *EGF* resulted in independent OS prognostic factors. *EGF* was confirmed as an independent factor along with TNM stage in HER2-overpressed mGC from TCGA collection. Our study highlighted factors involved in the WNT/β-catenin pathway that interconnected E-CAD with *HER2* overexpression and patient survival.

## 1. Introduction

The human epidermal growth factor receptor 2 (*HER2*, also called *ERBB2*), is a member of the HER family of tyrosine kinase receptors, which includes *EGFR*, *HER3*, and *HER4*. The *HER2* functions as a stabilizer of homodimers and heterodimers of ligand bound HER receptor family members, leading to enhancing downstream mitogen and proliferative signals. The extracellular domain (ECD) of *HER2* may be cleaved by proteases, such as the matrix metalloproteinases (MMPs) and the disintegrin and metalloproteinases (ADAMs), and then released in the serum as a soluble form (sHER2). The truncated *HER2* form, which remains membrane-associated, is found to be 10-to 100-fold more active than the full-length protein *HER2*. *HER2* gene amplification or protein overexpression (i.e., tumors scored 3+ by IHC), account for about 10% to 22% of gastric cancer (GC) and represent a requisite for the addition of trastuzumab (anti-HER2) to first-line chemotherapy in unresectable or metastatic GC (mGC) since its addition increased survival from 11.1 to 13.8 months versus chemotherapy alone [1].

Cadherin 1 *(CDH1)* gene encodes for E-cadherin (E-CAD), a cell-adhesion molecule involved in maintaining epithelial tissue integrity. E-CAD reduction is associated with epithelial–mesenchymal transition (EMT), generation of stem-cell, and metastasis [2]. Moreover, a germline pathogenic variant of the *CDH1* gene is a distinctive characteristic of hereditary diffuse gastric cancer (HDGC) syndrome. In a previous study, we observed an increased overall survival (OS) in treated HER2-positive mGC patients having the rs16260A-rs1801552T *CDH1* variants, compared with patients presenting other *CDH1* variants [3]. The first variant, the rs16260 C > A single nucleotide polymorphism (SNP) that is located −160 bp upstream of the transcriptional start site of the *CDH1* gene, was linked to an altered *CDH1* promoter activity, resulting in a decreased mRNA *CDH1* stability and gene transcription efficiency [4,5]. The second variant, the rs1801552 C > T, is located in the exon 13, near the poly-A signal, a position that has been proposed to cause instability in the mRNA *CDH1* sequence or alteration in the extracellular domain involved in cell–cell adhesion [6,7].

The relation between *HER2* overexpression and *CDH1* alterations and their effect on patient survival is unknown.

In the present study, we proved that *CDH1* mRNA expression was specifically associated with HER2-overexpression. Additionally, investigating the molecular mechanism linking *HER2* upregulation with mRNA *CDH1* and E-CAD expression, we found in the canonical wingless-type (Wnt)/β-catenin pathway the joining link. Moreover, *EGF, RARA, RPL19*, and *CTNND1* mRNA expression that we found involved in the Wnt/β-catenin signature were identified as valuable markers for HER2-positive mGC patient survival. In the second part of the study, we focused our attention on the co-expression of *HER2, RARA*, and *RPL19*, which have been known to colocalize at the chromosome 17q region. We hypothesized, according to the established role of ribosomal proteins in mediating response to nucleolar stress [8], and *RARA* on β-catenin regulation [9] a role of these genes to impact on OS in HER2-positive GC.

## 2. Materials and Methods

### 2.1. Study Population

We collected tissues from two series of patients. Series 1 consisted of 38 patients with histologically proven mGC admitted from 2012 to 2017. Patients received chemotherapy or chemotherapy plus Trastuzumab according to HER2-positive status (IHC 3+ or IHC 2+/fluorescence or silver in situ hybridization (ISH)-positive). The clinicopathological characteristics of these patients are shown in Table 1. Overall survival (OS) from the time of the diagnosis of GC (OS-GC) and the metastasis (OS-mGC) were collected. OS duration was calculated by the difference of GC diagnosis and metastasis to death or last follow-up, with no restriction on the cause of death.

Series 2 consisted of 36 patients with a histologically confirmed GC or mGC diagnosis ascertained from 2011 to 2018. The clinicopathological characteristics of these patients are shown in Table 1. OS from the time of GC diagnosis was collected.

Ethical approval for this study was obtained from Comitato Etico Unico Regionale FVG (approval number CRO 2019-03). The study was performed under the 1964 Helsinki Declaration and its later amendments. Patient consent was obtained.

A third series generated by the TCGA Research Network was used as a validation set [10] (https://www.cancer.gov/tcga, accessed on 3 May 2021; 2017); and licensed under the Creative Commons Attribution-ShareAlike 3.0 International License. The data released do not require informed patient consent. Data included 354 GC cases. RNA expression levels of the investigated genes (expressed as fragments per kilobase million (FPKM)) in tumor tissue and time of death or last follow-up were collected for every single patient. Patients with ≥80th percentile of *HER2* expression were considered as HER2-overexpressing cases. TCGA patients with clinical survival data were classified according to the optimal cut-off FPKM value of each selected gene into low-survival (LS) and high-survival (HS) groups by the TCGA Research network. Table 1 lists the best cut-off value and log-rank test *p*-value by comparing the prognostic risk of the two groups for each gene.

### 2.2. Design of the Study

We analyzed the mRNA profile of 38 samples from Series 1 (according to their *HER2* status) and 36 samples from Series 2 (according to their metastatic status) using the NanoString technology. To this aim, we designed a custom NanoString panel of 44 genes (Appendix A Table A1) according to genes associated with molecular signature to classify GC [11] and E-CAD-catenin complex [12] reported in the literature. Tumor specimens were collected at GC diagnosis before treatment.

Seeking to evaluate the role of E-CAD in the context of HER2-positive GC, the study design cross-analyzed genes differentially expressed between Series 1 and Series 2 and between Series 1 and Series 3. The hypothesis is that significant differences in mRNA gene expressions found in both Series 1 and Series 2 may more likely be associated with a generic metastatic process not directly dependent upon HER2-overexpression. Moreover, if the best cut-off for the same gene was able to discriminate a patient’s survival in Series 3, we assumed that this gene may affect the patient’s survival.

### 2.3. RNA Extraction and NanoString Quantification

Total RNA was extracted from 2–3 sections of 5–10 μm thick FFPE sections using the RNeasy DSP FFPE Kit (Qiagen, Hilden, Germany) according to the manufacturer’s protocol. DNase for optimized removal of eventual genomic DNA contamination was used. RNA quality was assessed using the High Sensitivity RNA Screen Tape kit (Agilent, Santa Clara, CA, USA)) on the 2200 Tape Station system (Agilent, come prima). The concentration of extracted RNA was found using the Qubit™ RNA HS Assay (Life Technologies Corporation, Eugene; OR, USA) on the Qubit fluorometer 2.0. instrument (Invitrogen idem). The samples with RNA concentrations of <40 ng/μL or absorbance A260/A280 ratios <1.5 were considered inadequate and were excluded from the analysis. Two hundred and fifty ng of total RNA extracted was hybridized overnight at 65 °C with probes. A custom NanoString panel was designed, including 44 previously published genes representing molecular mGC subtypes or CDH1-related gene signatures. The list of genes included in the panel and incorporating three housekeeping genes (B2M, GAPDH, HPRT1) as controls were reported in Appendix A Table A1. A NanoString nCounter Analyzer System (NanoString Technologies, Seattle, WA, USA) was used to count the number of RNA transcripts according to the manufacturer’s procedures. Raw data were then normalized for each tumor case by using the geometric mean of the positive controls and by subtracting the background level calculated as the geometric mean plus 2 standard deviations of the counts of the housekeeping genes included in the assay using the nSolver 4.0 software (NanoString Technologies, Seattle, WA, USA). Normalized data were Log2-transformed and fitted to a linear model for further analyses.

### 2.4. Cell Line Assay

The HER-2 overexpressing NCI-N87 gastric cell line was obtained from the American Type Culture Collection (ATCC, Manassas, VA, USA) and cultured according to the recommended specifications.

For the experiments, cells reaching 90% of confluence were detached with TrypLE Express (Life Technologies Corporation, Grand Island, NY, USA) following the manufacturer’s instructions, plated, and then cultured in flat-bottomed 12-wells plates for 5 days. The effect of recombinant human EGF (Bio-techne, Minneapolis, MN, USA) was tested by changing the culture medium with a fresh medium containing EGF at concentrations of 25, 50, and 100 ng/mL and culturing cells for 24 h. The effect of EGF plus Trastuzumab (Ontruzant) (Organon Italia, Roma, Italy) was evaluated by adding 5 ug/mL Trastuzumab to cell cultures treated with EGF at the same concentrations and conditions mentioned above.

After 18 h of further incubation, cells were collected and stored at −80 °C. RNA was extracted using the RNeasy Plus Micro kit (Qiagen, Hiden, Germany), according to the manufacturer’s protocol. RNA quantification was performed using Nanodrop (Thermo Fisher Scientific, Wilmington, DE, USA).

Nanostring analysis was then performed as reported at point 2.3.

### 2.5. E-CAD and HER2 Histological Evaluation

A formalin-fixed, paraffin-embedded FFPE tumor block was cut into 4-μm-thick sections for hematoxylin and eosin (H&E) immunostaining. Immunohistochemistry was performed by using the antibodies for *HER2* (clone 4B5, Ventana Medical System, Tucson, AZ, USA) and E-cadherin (clone NCH-38; Dako, Carpinteria, CA, USA). The E-CAD staining was scored on three scales: negative/weak staining (score 0) when less than 10% of tumor cells with strong IHC intensity (2–3+) or less than 30% of cells with weak intensity (1+) were present; reduced staining (score 1) when 10% to 90% of cells showed a strong IHC intensity or about 30% showed a weak intensity; normal staining (score 2) when more than 90% of cells showed a strong IHC intensity.

### 2.6. Serum Sample Collection for Soluble HER2 (sHER2) and Soluble E-CAD (sCDH1) Evaluation

We measured the concentration of serum sE-CAD and sHER2 levels before treatment in 55 patients with mGC: 4 patients from Series 2 and 51 patients for whom we had collected serum but a tissue specimen was not available. Among the 55 patients with mGC, 6 had overexpressed *HER2*. A second serum sample 3 weeks after starting the therapy was tested in 85.7% (42/49) of patients who showed metastases and in 83.3% (5/6) of patients with HER2-positive GC. Two independent groups of 44 blood donors (BD) and 49 GC patients without metastasis, with 14 of them included in Series 2, were tested to compare the median of serum sHER2 and sCDH1 levels in the general population and GC before treatment. Analysis was performed on patients whose consent was provided. Serum levels of the sHER2 were estimated using the human *ErbB2* (*HER2*) ELISA kit (Thermo Scientific, Invitrogen, MA, USA), according to the manufacturer’s instructions. Serum levels of the E-CAD (sE-CAD) were estimated using the human E-CAD EIA kit (Thermo Scientific, idem), according to the manufacturer’s instruction. All assays were performed in triplicate.

### 2.7. DNA Isolation and *CDH1* Genotyping Assay

Genomic DNA was extracted from each subject’s peripheral blood lymphocytes using an EZ1 DNA blood kit (QIAGEN, Hilden, Germany) according to the manufacturer’s instructions. Genotype analysis of two *CDH1* gene variants, rs16260 SNP, and rs1801552 SNP, associated with patients’ survival was performed by PCR as previously reported [3]. After amplification, the PCR product was sequenced using the Big Dye v3.1 Terminator Cycle Sequencing Kit (Life Technologies, Monza, Italy) with the Applied Biosystems 3130 automated sequencer (Applied Biosystems, Foster City, CA, USA). The sequence data were aligned and analyzed using the Codon Code Aligner software (CodonCode Corporation, Centerville, MA, USA).

### 2.8. Statistical Analysis

Individual gene sets were divided into *HER2* and metastatic signature groups. The normalized log_2_-transformed mRNA expression data of cases were analyzed by univariate and multivariate regression tests. High and low expression of genes in the subtypes were generated and tumors were categorized based on these expression patterns. Gene expression levels were correlated against *HER2* and *CDH1* IHC categories using independent samples *t*-test. Serum sHER2 and sCDH1 concentrations between cases and controls, and between before and 3 weeks after starting therapy, were compared using Student’s *t*-test. The significance of differences between groups with a nonparametric data distribution was analyzed with the Mann–Whitney U test for two independent groups. Univariate and multivariate analyses concerning expression data to predict the effect on OS were performed using a Cox proportional hazards regression model. OS was defined as the duration from the date of GC diagnosis (OS-GC) or the diagnosis of metastasis (OS-mGC) to death or last follow-up, with no restriction on the cause of death. A *p*-value < 0.05 was considered significant. Analyses were undertaken using the MedCalc Statistical Software, version 19.0.4 (MedCalc Software bvba, Ostend, Belgium).

## 3. Results

### 3.1. Patients’ and Series Characteristics

Overall, the mRNA expression profiles of 74 FFPE GC tumor samples collected before treatment were analyzed. Samples were grouped into two series. The first series included 38 mGC 13 of which HER2-positive and 25 HER2-negative. The second series included 36 HER2-negative GC, 10 of which were metastatic at the diagnosis, and 26 of which were locally advanced. A third series included data of 354 cases of GC retrieved from the Cancer Genome Atlas (TCGA) consortium that correlated gene expression and survival analysis. The clinicopathological characteristics of patients are reported in Table 1.

### 3.2. Analysis of the Impact of HER2 and E-CAD status on OS of Patients

**Figure 1:**
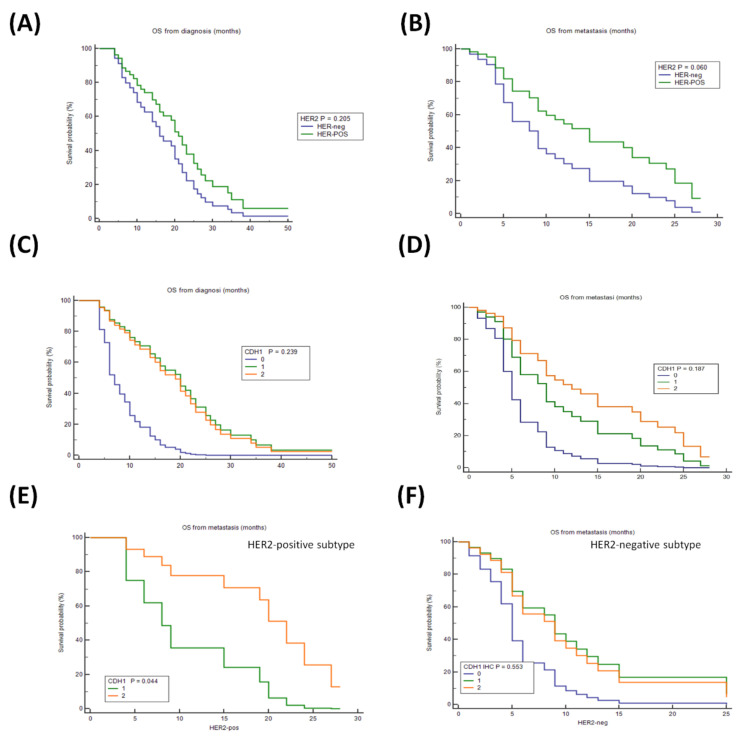
Cox proportional regression curves for OS (Series 1, *n* = 38). The predictive power of *HER2* (**A**,**B**) and *CDH1* (**C**,**D**) signatures at the time of diagnosis and at the time of the diagnosis of metastasis, respectively. The predictive power of *CDH1* signature in (**E**) HER2-positive mGC and (**F**) HER2-negative mGC subtypes.Log-rank *p*-values and hazard ratio (HRs) were calculated.OS at the diagnosis of GC: (**A**) HER2-negative mGC (HER-neg): reference category; HER2-positive mGC (HER-POS): HR 0.64 (95%CI 0.32–1.29), *p* = 0.205. (**C**) E-cadherin expression (*CDH1* score 2): reference category; E-cadherin reduced expression (*CDH1* score 1): HR 0.91 (95%CI 0.45–1.84), E-cadherin low expression (*CDH1* score 0): HR 4.54 (95%CI 0.94–21.83), *p* = 0.060. OS at the diagnosis of metastasis: (**B**) HER-neg: reference category; HER-POS: HR 0.51 (95%CI 0.25–1.05), *p* = 0.07. (**D**) E-cadherin (*CDH1* score 2): reference category; E-cadherin reduced expression (*CDH1* score 1): HR 1.60 (95%CI 0.81–3.17); E-cadherin low expression (*CDH1* score 0): HR 3.71 (95%CI 0.80–17.23), *p* = 0.09. OS at time of the GC diagnosis HER2-positive mGC: (**C**) *CDH1* (score 2) reference category; *CDH1* reduced (score 1) HR 4.11 (95%CI 1.05–16.11), *p* = 0.04. (**D**) *CDH1* (score 2) reference category; *CDH1* reduced (score 1) HR 1.12 (95%CI 0.49–2.57), *p* = 0.79; *CDH1* low (score 0) HR 2.60 (95%CI 0.54–12.41), *p* = 0.23.

After stratification of patients by *HER2* status in Series 1, no significant survival differences were found considering the OS-GC (HER2-negative: median OS 15 months (95%CI 10–38); HER2-positive: median 20 months (95%CI 8–50) (Figure 1A)) while a slight increase in median OS-mGC, although not statistically significant, was found in HER2-positive group (median: OS: 16 months (95%CI 6–28)) compared with the HER2-negative cases (median OS: 8 months (95%CI 5–25) (Figure 1B)). After stratification by E-CAD, no significant OS survival differences were found between patients with low E-CAD (score 1; median OS 15 months (95%CI 7–50)) compared with those with high E-CAD expression (score 2; 19 months (95%CI 14–34)); 2 patients with a complete lack of E-CAD staining showed a lower OS (score 0; median OS 6 months (95%CI 6–10) (Figure 1C)). A similar trend was obtained by analyzing the OS-mGC (E-CAD score 0: median 4 months (95%CI 4–8); E-CAD score 1: 9 months (95%CI 5–25), E-CAD score 2: 11 months (95%CI 5–28) (Figure 1D)). Overall, patients with HER2-positive GC and high E-CAD expression (score 2) showed better survival, although these findings did not reach statistical significance.

On these grounds, the effect of E-CAD expression was evaluated according to the *HER2* status. Interestingly, high E-CAD expression had a different weight and significantly improved the survival of patients with HER2-positive compared with HER2-negative mGC (HER2-positive: E-CADscore 2: median OS 19 months (95%CI 2 to 28); HER2-negative: E-CADscore 2: median OS: 6 months (95%CI 3 to 25) (Figure 1E,F)). Moreover, while a reduction of E-CAD (score 1) was associated with worsening survival in HER2-positive mGC (HER2-positive: E-CADscore 1: median OS 9 months (95%CI 4–20); E-CADscore 2: median OS 19 months (95% CI 2–28); *p* = 0.044) (Figure 1E)), no statistically significant differences in HER2-negative mGC were found (Figure 1F). Overall, these data showed that higher E-CAD expression was associated with an increase in OS in HER2-positive mGC.

### 3.3. Comparison between IHC versus mRNA for Detecting HER2 and E-CAD

We assessed the concordance between IHC and mRNA quantification measured by Nanostring of *HER2* and E-CAD. Cases above the 80th percentile for *HER2* mRNA expression level (2203 (95%CI 1062–11,648)) showed over 86% (33/38) match with cases classified as HER2-positive (i.e., IHC3+, or 2+ and ISH+). Cases above the 80th percentiles for *CDH1* (1770 (95%CI 1499–2381)) showed about 79% (30/38) match with E-CAD IHC expression. High consistency between IHC (and/or ISH) and mRNA expression data was thus found either for *HER2* (*p* < 0.001, Supplementary Figure S1A) or for E-CAD (*p* = 0.039, E-Cad score Jonckheere trend test, *p* = 0.035, Supplementary Figure S1B).

### 3.4. Serum Soluble E-CAD Levels Are Reduced after Treatment Both in HER2-Negative and in HER2-Positive mGC

E-CAD may be released in the serum as sE-CAD form. sE-CAD had been identified as a pro-neoplastic inflammatory signal [13] and demonstrated to be a powerful ligand of different members of the EGFR family [14]. We evaluated sE-CAD levels in the serum of 44 blood donors (BD) and 104 patients with GC. The sE-CAD concentration in BD, and prior any treatment in non mGC (n = 49) was similar (median: 4187 pg/mL and 3033 pg/mL, respectively). Compared with non-metastatic GC, the concentration of sE-CAD in mGC before any treatment increased in HER2-negative cases (n = 49; median 4885 pg/mL, *p* < 0.05) and much more in HER2–positive cases (n = 6, median 6001 pg/mL, *p* < 0.05) (Supplementary Figure S2). The concentration of sE-CAD decreased after treatment in both the mGC groups (difference prior to and after treatment: HER2-negative: −2541, (95%CI −3567 to −1100); HER2–positive: −3667 (95%CI −5791 to −639), *p* < 0.05). Results indicated that a higher sE-CAD level was likely associated with metastasis, with a higher median level in HER2-positive cases, and that the concentration significantly decreased in the overall metastatic groups after treatment.

### 3.5. CDH1 rs16260 Genetic Variant Associated with E-CAD in HER2-Positive MGC

A two-way A analysis was used to estimate simultaneously how the median *CDH1* mRNA level changed according to IHC E-CAD expression (IHC score 0–1 to 2) and *CDH1* SNPs (rs16260-A, Supplementary Figure S3A) and rs1801552-T Supplementary Figure S3B). Similarly, we evaluated how the median *HER2* mRNA changed according to *HER2* status and the presence of the *CDH1* SNPs (rs16260-A, Supplementary Figure S3C) and rs1801552-T Supplementary Figure S3D).

In patients carrying the rs16260-A variant (genotype A/A + A/C), the median concentration of *CDH1* and *HER2* mRNA was found higher in cases showing high E-CAD expression (score 2) (Supplementary Figure S3A) and in patients with HER2-positive mGC (Supplementary Figure S3C), respectively.

We did not find significant evidence for a change in *CDH1* and *HER2* mRNA levels in patients carrying the rs1801552-T variant (Supplementary Figure S3B,D).

Of note, the number of patients with rs16260 A variant was higher in the HER2-positive than in HER2-negative mGC patients (11 of 24, 45.8% and 7 of 24, 29.1%, respectively) Supplementary Figure S3E. On the other hand, in HER2-negative mGC, patients having the wild type *CDH1* (rs16260 C/C) showed a reduced survival compared to patients with HER2-positive Supplementary Figure S3F.

We next evaluated and compared the NanoString gene expression panel according to the *CDH1* rs16260 variant; results revealed increased levels of WNT3, *RARA*, Src, and a reduction of the MMP2 concentration in patients carrying the *CDH1* rs16260 A-variant (Supplementary Table S2, Mann–Whitney test).

### 3.6. Genes Differentially Expressed according to the HER2 Status

Looking to find genes differentially expressed according to *HER2* status we cross-analyzed mRNA levels between Series 1 and Series 2 and between Series 1 and Series 3.

The differences in mRNA expression among groups were presented in Table 2. Data were listed in a *p*-value increased order obtained from Series 1 (*t*-test). The list for genes specifically associated with HER2-positive mGC included *RARA*, *MLH1*, *EGF*, *ZEB1*, *VCL*, *RXRA*, *ATM*, *RPL19*, *WNT1*, *KLRG1*, *MDM4*, and *SNAIL2*. Genes as *TP53*, *SRC*, *SNAIL1*, and *MSH6* were considered not specific for HER2-positive GC because they were also found differentially expressed in HER2-negative GC cases according to the metastatic status (Series 2, Table 2). Several genes associated with *HER2* overexpression—i.e., *RARA*, *MLH1*, *EGF*, *ZEB1*, *VCL*, *RXRA*, *WNT1*, *KLRG1*, and *SNAIL2*—were also differentially expressed in Series 3 (*p*-value for survival <0.05, Table 2) suggesting their potential association with GC patient’s survival in the general population.

### 3.7. Wnt/-β-Catenin Signature Joined CDH1 and HER2 mRNA Expression

**Figure 2:**
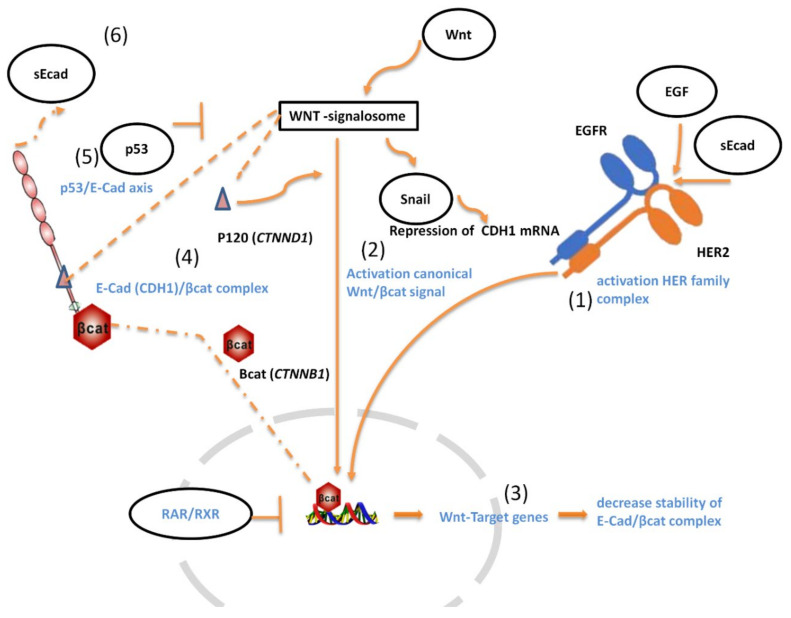
Functional interpretation of the relationship between *HER2* and *CDH1* based on gene expression data obtained by NanoString analysis in a mGC patient cohort (**1**) *EGF* is one of the most overexpressed mRNA genes in HER2-positive mGC (*p* = 0.0119, Serie 1, Table 2). The binding of EGF ligand to the EGFR/HER2 complex is known to activate β-catenin signaling by either promoting the release of β-catenin from the cytoplasmic membrane or disrupting the β-catenin destruction complex [15,16,17]. (**2**) Canonical Wnt/β-catenin signaling cascade is initiated by the binding of several Wnt (s) ligands to the signalosome-Wnt/receptor complexes, which allows β-catenin (*CTNNB1*) translocation into the nucleus and transcription of *Wnt* target genes. Wnt1, a specific ligand that activates the canonical Wnt/β-catenin signal, is overexpressed in HER2-positive mGC patients (*p* = 0.0282, Table 2). Upon Wnt activation, the activity of GSK-3β, a component of the Wnt signalosome, is inhibited, leading to an increased accumulation of *SNAIL* [18]. We found an increased level of *SNAIL* mRNA, one of the most repressors of E-CAD/*CDH1* expression, in HER2-positive mGC (*p* = 0.0399, Table 2). (**3**) Many target genes of Wnt signaling have been reported to prevent E-CAD expression [19,20]. The RAR complexes (*RAR/RXR* heterodimers) compete with the WNT-signalosome for β-catenin degradation and β-catenin-mediated gene transcription inhibition [21]. In HER2-positive mGC, we observed a downregulation of *RXRA* (*p* = 0.0255, Table 2), but *RARA* was overexpressed (*p* = 0.0080, Table 2). Of interest, we demonstrated that *RARA* expression correlated with *HER2* mRNA level (Figure 3), possibly due to the *RARA* and *HER2* gene co-localization. Thus, *RARA* expression can be modulated by the variation of the length of the *HER2* amplification, leading to an overall modulation in the *RAR/RXR* inhibition of the β-catenin targeted genes. (**4**) Destabilization of p120-cadherin (*CTNND1*) from E-CAD complex also increases the *Wnt/β-catenin* signaling by forming a complex protein interaction with the WNT-signalosome at the cellular membrane [22,23]. The difference of *CTNND1* expression between HER2-positive and HER2-negative mGC expression is quite significant in our series (*p* = 0.0583, Table 2). (**5**) Reduction of *TP53* mRNA expression is strongly associated with HER2-positive mGC (*p* = 0.0034, Table 2). P53 is known to be a suppressor of canonical Wnt signaling [24]. Moreover, p53 had been recently reported as a necessary factor for *CDH1* mRNA transcription through its interaction at the *CDH1* promoter region [25]. (**6**) The proteolytic cleavage of the extracellular domain of E-CAD releases the soluble sE-CAD form, which can activate the EGFR downstream signaling competing with EGF [14]. The sE-CAD concentration was found to be higher in HER2-positive mGC patients and its level decreased after treatment with trastuzumab (Supplementary Figure S2a).

Genes found differentially expressed in Series 1 according to their role in signature pathways were illustrated in Figure 2. The analysis revealed the canonical Wnt/β-catenin as a pathway that correlated well with *CDH1* mRNA and *HER2* mRNA expression. Three other signatures resulted related to HER2-overexpression: the RAR/RXR, p53, and KLRG1 pathways.

*HER2* has no known ligand and functions as a heterodimer linking other members of the *HER2* receptor family, i.e., EGFR, HER3, and HER4. Specifically, we found that the stronger ligand of EGFR, the epidermal growth factor (EGF), was over-expressed in the HER2-positive mGC group (*t*-test *p* = 0.0119, Table 2). Upon binding to EGF, EGFR can dimerize, thus consenting to the activation of the EGFR/HER2 complex [26]. Binding to EGF produces the phosphorylation of specific tyrosine residues in the cytoplasmatic EGFR tail that are needed to send signals for cell survival and proliferation. In addition, EGF-EGFR binding also permits the formation of the docking site for the c-Src protein [27], which in turn mediates the EGFR phosphorylation necessary to the internalization of the complex [28]. Overall, EGFR activates c-Src, and it further increases the response of EGF/EGFR-activation. c-Src plays a key role in multiple signaling pathways [29], including the disruption of the binding of β-catenin (CTNNB1) to cadherin and loss of cadherin complexes from the cell surface by p120 (CTNND1) phosphorylation [30,31].

Single members of the wingless-type (Wnt) family bind specific frizzled receptors (FZD) together with the low-density lipoprotein receptor-related Protein 5/6 (LRP5/6), to activate the canonical Wnt downstream signal [32]. Although the family of Wnt genes was lowly expressed, we detected an increased expression of *WNT1* (*t*-test *p* = 0.0282; Table 2) in HER2-positive mGC.

**Figure 3:**
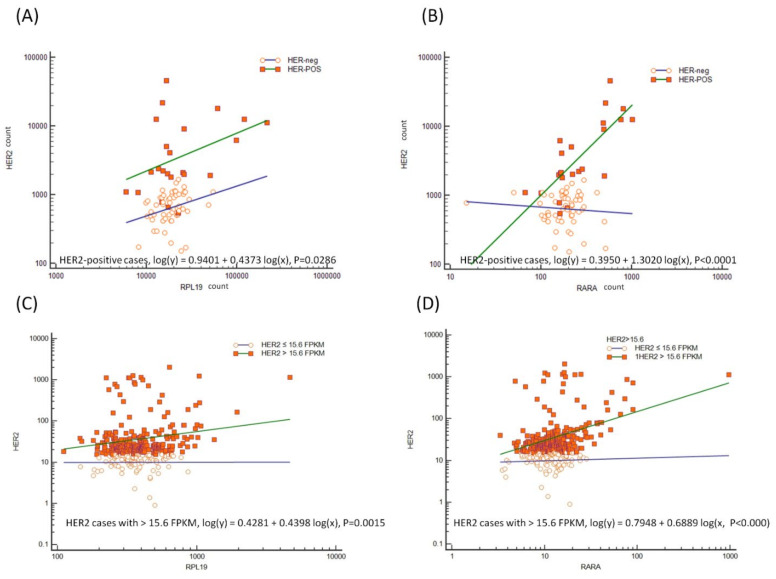
Evaluation of *HER2* mRNA concentration in terms of *RPL19* (**A**–**C**) and *RARA* (**B**–**D**) concentrations (linear regression analysis). The increase in *RPL19* and *RARA* mRNA concentration is associated, with a positive HER2-positive status in both our series (**A**,**B**) and patients extrapolated from the TCGA data set (**C**,**D**). 15.6 FPKM corresponded to the 80th percentile of *HER2* expression in the TCGA data set. TCGA, Cancer Genome Atlas (https://www.cancer.gov/tcga, accessed on 3 May 2021).

We also found a different expression of components of the retinoic acid (RA) signaling pathway. The RAR pathway normally upon stimulation inhibits the Wnt/β-catenin pathway by affecting β-catenin function thus affecting the cell cycle arrest control and the differentiation of target cells like stem-cells [33,34]. In HER2-positive mGC cases, we found *RARA* over-expressed but *RXRA* mRNA under-expressed (*p* = 0.0080; *p* = 0. 0255, respectively, Table 2). Moreover, a direct relationship between *RARA* and *HER2* mRNA expression (*p* < 0.0001, Figure 3A), whose genes are both on the same chromosomal 17q region, was found and this finding was confirmed in the TCGA dataset (*p* < 0.0001, Figure 3B). Of interest, mRNA expression of *RPL19* gene that is localized on the same 17q region of *HER2*, was also positively correlated with *HER2* overexpression either in our series or in the TCGA data set (Figure 3C,D).

Moreover, we detected a lower expression of the inhibitory Killer Cell Lectin-like Receptor (KLRG1) in HER2-positive mGC (*p* = 0.0290) (Table 2). KLRG1 is an inhibitory receptor presented by natural killer (NK) and T-cells to inhibit and protect the ‘healthy’ cell from the immune response [35]; its natural ligand is E-CAD expressed on the cell surface. Some alterations of genes involved in the p53 signaling, i.e., *MLH1*, *ATM*, *RPL19*, *MDM4*, and *MSH6* were found differentially expressed in HER2-positive compared with HER2-negative cases (Table 2, Series 1). P53 is activated in response to DNA damage [36], and as more recently reported also to nucleolar stress [37,38]. Results reported in Table 2 showed that HER2-negative mGC in Series 2 showed a significant increase in *TP53* (*p* = 0.028) and *MSH6* (*p* = 0.036) mRNA expression, while, both genes showed a lower mRNA expression in HER2-positive mGC compared with those of HER2-negative (Series 1, *p* = 0.0034, and *p* = 0.0486). Overall, these results suggested that the involvement of p53 signaling may be differentially modulated in mGC based on their *HER2* status.

### 3.8. External Validation of Genes Associated with CDH1 and HER2 Overexpression in the TCGA Dataset

We explored if our model fit in another series that incorporated gene expression information. To this aim, we considered cases with high *HER2* expression extrapolated from the TCGA data set (FPKM ≥ 80th percentile; 80th = 37.61 (95%CI 34.70 to 42.43) FPKM). Seventy-one patients (20.1%) showed a *HER2* expression ≥80th percentile. Results of Mann–Whitney analysis (Supplementary Table S3) confirmed the association of genes *RPL19, SRC, KLRG1, WNT1, RARA, ZEB1, SNAI2*, and *EGF* with the ≥80th percentile of *HER2* expression. A STRING protein–protein interaction network covering genes was found associated with mRNA HER2-overexpressed GC that shows evidence of their relationship (Supplementary Figure S4).

### 3.9. Combination of Trastuzumab with EGF-Induced *CDH1* mRNA Expression in NCI-N87 Gastric Cell Line

**Figure 4:**
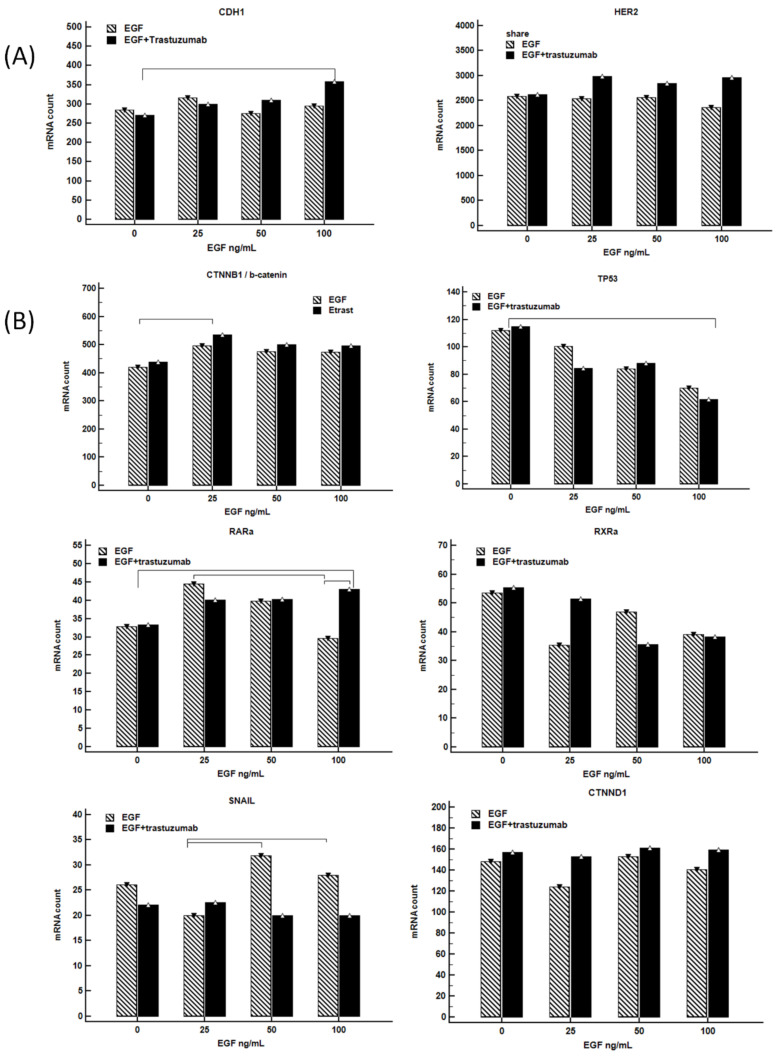
Column graphs of gene mRNA expression in NCI-N87 cell line treated with a dose-escalation of EGF (0 to 100 ng/mL) with/without trastuzumab (5 ug/mL). Following exposure to EGF for 24 h, and a further 18 h of incubation with/without trastuzumab, RNA was extracted from the NCI-N87 and used for NanoString analysis. The median mRNA count for each gene at the different treatment conditions was expressed as a column graph. The results were reported as a connector line when statistically significant (*p* ≤ 0.05). Combination of trastuzumab with EGF-induced *CDH1* mRNA expression in NCI-N87 gastric cell line (**A**). Results reported support the model of canonical Wnt/β-catenin signature (**B**). Overall treated cells show an increased CTNNB1/β-catenin versus the control without EGF (*p* = 0.012); a reduction of *TP53* *p* = 0.009. Moreover, cells treated with only EGF showed an increased *SNAIL* level compared to the control without treatment (*p* = 0.006).

The potential activity of trastuzumab (5 ug/mL) with the increasing-dose combination of EGF (0, 25, 50, 100 ng/mL) on *CDH1* and *HER2* mRNA expression was evaluated in the HER2-positive human gastric NCI-N87 cell line (Figure 4A). The level of *CDH1* mRNA did not change significantly in controls and cells treated with EGF, except in the case of cells treated with the lowest EGF concentration (25 ng/mL). However, the expression of *CDH1* was upregulated proportionally in cells treated with an increased concentration of EGF plus trastuzumab. The addition of trastuzumab produced a slight augmentation of *HER2* mRNA expression levels in all treated samples.

In addition, we performed ANOVA pair tests to identify differentially expressed mRNA related to the Wnt/-β-catenin signature that joins *CDH1* with *HER2* mRNA expression (Figure 4B). Analyses revealed that both EGF and EGF plus trastuzumab treatments correlated with a slight increase of CTNNB1/β-catenin and a significant reduction of *TP53* expression level (*p* = 0.012, and *p* = 0.009, respectively). EGF plus trastuzumab treatment led also to a reduction of *RXRa* level, although the difference did not reach a statistical significance, while the detrimental reduction of *RARa* expression in cells treated with EGF was counterbalanced by the anti-HER2 treatment. Data also demonstrated an augmented *SNAIL* mRNA production in NCI-87 cell treated with EGF at ≥50 ng/mL concentrations but a strong reduction after the addition of trastuzumab (*p* = 0.006).

### 3.10. Identification of Genes Associated with Wnt/-β-Catenin Signature for Discriminating OS

We stratified HER2-positive mGC patients (n = 13) of series 1 into low- (LS) and high-survival (HS) groups according to the median time of survival to investigate if one of the genes associated with *HER2* overexpression may also affect the survival ((from GC diagnosis median OS 22 months (95%CI 12 to 27); from metastasis median OS: 19 months (95%CI *7* to 23)). Seven patients resulted in LS and 6 in the HS group. Table 3 summarizes the results of the receiver operating characteristic (ROC) curve analysis that estimated for each gene the specificity to discriminate between LS and HS groups. The analysis indicated the *EGF*, *CTNND1*, *SRC*, *TP53*, *SNAIL1*, *RPL19*, and *RARA* as genes capable of discriminating between LS and HS at GC diagnosis. *SRC*, *TP53*, and *SNAIL1* were excluded from the subsequent analysis since they were found also associated with metastasis in the HER2-negative subtype and thus considered not specific to HER2-overexpression (Table 2, Series 2). *EGF*, *RARA*, and *RPL19* were also able to discriminate LS from HS cases from the time of metastasis (Table 4). Hazard ratios of OS with the cut-off value were reported for each gene by using univariate Cox regression analysis (Table 3) from the time of GC diagnosis and Table 4 from the time of metastasis. At the time of GC diagnosis *CTNND1* and *RARA*, were revealed as independent factors for OS by multivariate analysis (*p* = 0.007, *p* = 0.0211) and *EGF* at the time of the diagnosis of metastasis (*p* = 0.037) (Table 3 and Table 4).

**Figure 5:**
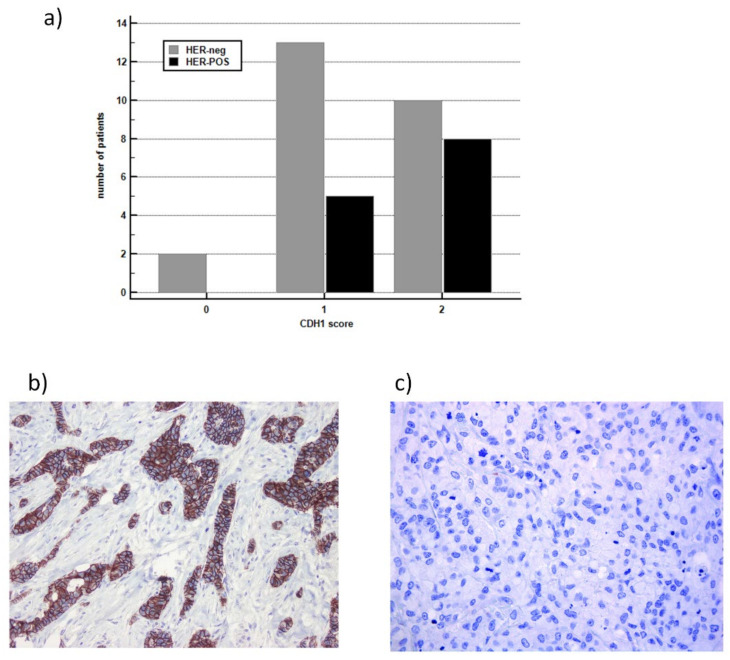
To better understand the role of *CDH1*/E-CAD expression in HER2-positive (HER-POS) mGC, we have used immunohistochemistry in formalin-fixed paraffin-embedded gastric tissues (*n* = 38). (**a**) In the HER-POS group, no patients showed missing E-CAD in the tumor cells (score 0), five patients showed a low E-CAD expression (score 1), and 8 patients showed a wide E-CAD expression (score 2); in the HER-neg, 2 patients had missed E-CAD, 13 patients had a low expression, and 10 patients showed a wide E-CAD expression in the tumor cells. HER2-POS compared to the HER-neg mGC patients showed a lower proportion of patients with a reduced expression of E-CAD (38.5% and 60.0%, respectively); however, due to the small sample size, the result did not reach statistical significance (Fisher’s exact test, *p* = 0.31). (**b**) Representative immunohistochemistry findings for E-CAD positive (**b**) and negative (**c**) in gastric tissue (40×).

To better understand the role of CDH1/E-CAD expression in HER2-positive (HER2-POS) mGC, we have used immunohistochemistry in formalin-fixed paraffin-embedded gastric tissues (*n* = 38). The number of patients with an increased E-CAD expression (score 0 to 2) is higher in HER2-positive compared to the HER2-negative mGC patients, as shown in the bar graph (Figure 5a). Figure 5b, c shows the representative immunohistochemistry findings for E-CAD positive (b) and negative (c) in gastric tissue, respectively.

**Figure 6:**
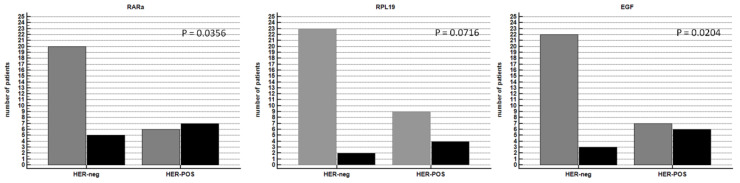
Distribution of patients with higher (black color) and lower (grey color) *RARa*, *RPL19*, and *EGF* mRNA expression than their respective mean level in HER2-positive (HER-POS) and HER2-negative (HER-neg) mGC. For all the analyzed genes, we found a higher mRNA expression level in the HER-POS compared to the HER-neg mGC patients; although, due to the small sample size, the result did not always reach statistical significance (Chi-square test, *p*-value reported in the figure).

To validate the results of Table 3 and Table 4, we analyzed the distribution of patients with higher (black color) and lower (grey color) *RARa*, *RPL19*, and *EGF* mRNA expression than their respective mean levels in HER2-positive and HER2-negative mGC. For all the analyzed genes, we found a higher mRNA expression level in the HER2-positive compared to the HER2-negative mGC patients (Figure 6).

**Figure 7:**
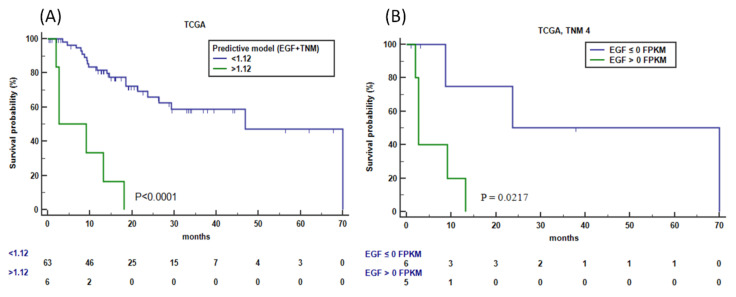
OS prognosis performance in TCGA data set. (**A**) OS patients’ survival according to the predictive model including *EGF* expression and TNM stage in HER2-overexpressed GC (HER2 > 15.6 FPKM). Samples were classified as low- and high-risk groups using coefficients of *EGF* and TNM stage with a mean cutoff score of 1.12 (Kaplan–Meier analysis). (**B**) Performance of *EGF* concentration in HER2-overexpressed (HER2 > 15.6 FPKM) mGC (*TNM4* stage) (Kaplan–Meier analysis). *TCGA*, Cancer Genome Atlas (https://www.cancer.gov/tcga, accessed on 3 May 2021).

To further validate the results, we used the TCGA dataset (Series 3), and stratified the 71 patients showing HER2-overexpression (FPKM ≥ 80th percentile) into low-survival (LS) and high-survival (HS) based on the median OS (46.9 (95%CI 18.47 to 70.00) months). The significant differences in gene expression between the two groups in univariate analysis were listed in Table 5. *RPL19, SRC, RARA*, and *EGF*, resulted in differentially expressed according to *HER2* overexpression and survival in both Series 3 and Series 1. Multivariate analysis retained EGF expression as an independent factor for OS. Inclusion of the TNM as a covariable in the multivariate analysis (stepwise search, *p* < 0.05 as cutoff) identified EGF and TNM stage 4 as two independent factors (coefficient = 0.317, *p* = 0.019 and coefficient = 1.084, *p* = 0.020, respectively). The resulting impact of EGF level plus TNM stage for OS according to the cut-off value of 1.12, as the mean of the predictive model, was shown in Figure 7A. Specifically, among patients with HER2-positive mGC (TNM-4, n = 11), patients having EGF concentration ≥0 FPKM showed a significantly shorter OS (median 2.7 months) compared with patients with EGF concentration <0 FPKM (23.8 months), with a hazard ratio of 7 (95%CI 1.33 to 37.37, *p* = 0.0217) (Figure 7B).

## 4. Discussion

In the present study, we delineate and personify the function of canonical Wnt/β-catenin signaling as a key pathway that correlates *CDH1* and *HER2* mRNA expression in mGC. The higher *CDH1* expression we found in cell line treated by a dose-escalation of EGF plus trastuzumab support this data. The identification of this pathway supports a previous study that showed a specific *CDH1* rs16260-A/rs1801552-T variant associated with a better prognosis in mGC [3]. Today, only validated biomarkers, such as *HER2* and MSI/PD-L1, are available to guide the treatment options in advanced GC [39]. However, due to the tumor heterogeneity of *HER2* expression and loss of *HER2* signaling following trastuzumab targeted therapy, the utility of HER2-positive as the only therapeutic choice is considered reductive and requires more in-depth study.

Hyperactivation of canonical Wnt/β-catenin signaling has been reported in various cancer types including about 30–50% of GC [40], but, for the first time, we associated this pathway with the HER2-positive mGC subtype and its survival. Indeed, mounting evidence has suggested that overexpression of Wnt/β-catenin signaling not only affects gastric cell proliferation, but also the GC relapse and metastasis by giving rise to a new cancer population of stemness-associated self-renewal cells [41,42]. Furthermore, the self-renewal cell activity was found to increase in HER2-positive cancer stem cells via Wnt/β-catenin signaling [43] that had also been implicated in important tumor growth processes such as cell nutrient acquisition, cell metabolism, and immune response against the tumor cells [44,45,46].

It is well known that canonical activation of the Wnt/β-catenin signature triggers the Wnt signalosome complex inactivation. Two scaffolds’ proteins: the Adenomatous Polyposis Coli (*APC*) and Axin, and two Ser/Thr kinases: the Casein Kinase 1 (*CK1*) and the Glycogen Synthase Kinase 3 beta (*GSK3β*) compose the Wnt signalosome complex, which targets β-catenin for proteasomal degradation. Upon Wnt stimulation, unphosphorylated β-catenin accumulated in the cytoplasm, and thus its migration to the nucleus is favored and leads together with the T cell factor/lymphoid enhancer transcription factors (*TCF/LEF*) causing WNT-response gene transcription and cell proliferation [29]. Furthermore, Wnt stimulation induces CK1-mediated p120 catenin and E-CAD dissociation from the Wnt-receptor complex, and then the signalosome vesicular internalization [36]. Overall, WNT1 binding to its receptor leads to both internalizations of the signalosome in endocellular vesicles and the cell membrane and consequently to the sequestration of the GSK3β from the cytoplasm, thus reducing β-catenin degradation and prognosis for patients with GC [47]. Although, in our series, no significant difference has been found in the β-catenin mRNA expression between HER2-positive and HER2-negative cases, this does not disagree with previous studies since most nuclear β-catenin protein accumulation is derived from the post-translational modification that occurs at the protein level, and interaction with other pathways—such as RAR/RXR-mediated signaling—thus is not necessarily expected to be seen at the mRNA level [48]. Intriguingly, β-catenin signaling represents a critical event for invasion and migration in a breast cancer model that overexpressed *HER2* [23], and β-catenin knockdown seems to sensitize cells to anti-HER2 treatment with trastuzumab [49]. Data analysis from NCI-N87 showed a slight quite significant increase of CTNNB1/β-catenin after EGF treatment that it was not abolished by the addition of trastuzumab, supporting this suggestion.

In the HER2-positive group from our series, *WNT1* species-specific via canonical Wnt/β-catenin pathway was overexpressed. Conversely, a difference in *WNT5* mRNA expression through the non-canonical Wnt/β-catenin pathway was not found; the result thus supports a specific contribution of canonical Wnt/β-catenin pathway in HER2-positive cases [50]. Previous studies had underlined the key role of Wnt1 signaling for the proliferation and progression of GC. Indeed, Seo Jaesung et al. had shown that the suppression of GPR177 (Wntless, WLS), an essential protein for the secretion of Wnt, suppressed tumor gastric proliferation [51]; while Suzuki et al., by using a specific model of stomach with a claudin-18.2 deficiency junction (stCldn18-KO) that reproduced the cancer Correa cascade in the absence of *H. pylori* infection, demonstrated that Wnt1 overexpression produced gastric tumors faster than stCldn18-KO without Wnt1 [52].

Our study also showed that poor patient survival in HER2-positive mGC was related to the loss of E-CAD mediated by the Wnt/β-catenin pathway. EGF was the best ligand of EGFR/HER2 heterodimer activation, and one of the most differentially expressed mRNA that we found associated with HER2-positive cases and poor survival in our series, like in the TCGA series. EGFR and Wnt/β-catenin crosstalk have been reported by many investigators [53,54]. Specifically, activation of *EGFR* family members (*EGFR, HER2*, and *HER3*), in an EGF-dependent manner, was demonstrated to cause by phosphorylation, the inactivation of the GSK3β kinase, and accumulation of the ß-catenin protein [31]. Moreover, both in GC as well as in breast cancer, several preclinical models have shown a relationship between the upregulation or activation of the *EGFR* and *HER3*, with the resistance to *HER2* [55,56,57].

Based on these results, an important part of drug research now combines drugs targeting both *HER2* and HER family members (e.g., bispecific antibodies, dual tyrosine-kinase inhibitor, pan-HER inhibitor) to improve patient survival and overcome the mechanism of resistance to trastuzumab [58,59,60]. The binding of EGF produces the phosphorylation of specific tyrosine residues in the cytoplasmatic EGFR tail that are required to transmit signals for cell survival and proliferation and the formation of the docking site for the c-Src protein [27,28,29], which consent to the internalization of the EGF/EGFR complex [28]. c-Src plays a key role in multiple signaling pathways [29], including the disruption of the binding of β-catenin to E-CAD and loss of the cadherin complexes from the cell surface by p120 (*CTNND1*) phosphorylation [30,31]. In our series, we found no differences in *SRC* mRNA expression between HER2-positive and HER2-negative cases, but SRC function is activated by phosphorylation and not necessarily associated with higher *SRC* mRNA expression.

*SNAI1* is a key regulator of suppressing E-CAD expression [61,62], and its expression is regulated by the Wnt cascade [63]. Consistently, we found that *SNAIL* was more expressed in the NCI-N87 cell line after treatment with a dose-escalation of EGF that activated the canonical Wnt cascade signaling, and that *SNAIL* expression was reduced by anti-HER2-treament. In HER2-positive mGC, we found that rs16260-A variant—located in the *CDH1* promoter region that interferes with the interaction of *SNAI1* and *CDH1* mRNA [4,5]—was associated with the E-CAD expression and with a better prognosis. Data confirm our previous study that showed an association between rs16260-A variant and a better prognosis in mGC [3]. In addition, we demonstrated a correlation between the rs16260 C > A variant with *CDH1* mRNA and with higher E-CAD expression (score 2) in HER2-positive mGC. Our data suggest a detrimental role of wild type *CDH1* in HER2-negative patients, which is abolished in HER2-positive mGC patients. We suppose that factors associated with the *HER2* status may interfere with the *CDH1* promoter region where the rs16260 variant is located; however, the limited number of cases studied did not consent a conclusive response.

P53 is known to be a suppressor of canonical Wnt signaling [24]. Moreover, p53 had been recently reported as a necessary factor for *CDH1* mRNA transcription through its interaction with the *CDH1* promoter region [25]. The gradual reduction of *TP53* expression after treatment of NCI-N87 cell line with a dose-escalation of EGF with/without trastuzumab is in accord with this model.

During GC progression, E-CAD is cleaved in part from the cell surface and released as a soluble sE-CAD form, which we found at a higher concentration in the metastatic group and a lower level in the same patients after treatment. Based on the knowledge that sE-CAD supports the HER-heterodimerization and activation [64] and that EGF promotes the shedding of sE-CAD [65], data support a relationship between E-CAD and response to *HER2* targeted therapy. Indeed, the activation of EGFR produces the detachment by SRC-mediated phosphorylation of the p120/E-CAD complex from the low-density lipoprotein receptor-related protein 5/6 (LRP5/6), which together with the frizzled receptors (FZD), activates the Wnt signalosome favoring the tumor cell growth [21]. Thus, HER2/EGFR activation by modulating the phosphorylation of E-CAD/βcatenin complex interferes with the sequestration of the Wnt signalosome and, in the same way, produces a reduction of E-CAD on the cell membrane and the release of sE-CAD that further may increase the EGFR signaling [22,46]. Moreover, serum sE-CAD/KLRG1 axis may inhibit KLRG1+ cytotoxic T-cells and NK cells function in the peripheral blood, probably suppressing their cytokine secretion and proliferation both in the periphery and in the tumor microenvironment. sE-CAD may then be proposed as a negative immune checkpoint able to reduce the effector function and number of anti-tumor immune cells in the tumor microenvironment. Overall, results support the hypothesis that *HER2* targeted therapy produces a reduction of *CDH1* mRNA in mGC, with a reduction in sE-CAD release and EGFR activation that leads to a reduction of the canonical wnt/β-catenin activation as a consequence. This model is in line with the hypothesis presented by Oh, D.Y. et al. [66] for which patients with low sE-CAD before anti-HER2 treatment could have a better prognosis.

Furthermore, the present study revealed the mRNA co-expression of *RPL19, RARA*, and *HER2* genes in patients due to their co-localization on the same 17q chromosomal region. *RARA* has a key role in the β-catenin function, and retinoid-based targets represent an important line of strategies for treating cancer [9]; while *RPL19*, a component of the 60S ribosomal proteins, is involved in the nucleolar structure with an impact on the regulation of p53 concentration [8]. Based on the possible co-expression of these genes in HER2-positive mGC, for the first time, we showed that both high *RPL19* and *HER2* mRNA and *RARA* and *HER2* mRNA expression were associated with a better OS.

## 5. Conclusions

Collectively, our data support an important relationship between canonical Wnt/β-catenin activation and poor prognosis in the HER2-positive mGC subtype. *HER2* overexpression is associated with Wnt-β-catenin activation and with an increase in *CDH1* mRNA production; however, sE-CAD and EGF through EGFR/c-Src activation and the difference of *SNAI1* interaction with the *CDH1* promoter region at the transcriptional level can regulate the final expression of E-CAD on the cell surface. In this context, both E-CAD expression and *EGF* concentration resulted prognostic OS factors in HER2-positive mGC. In addition, we found *RPL19* and *RARA* as co-expressed together with *HER2* mRNA, and they both in turn can interfere with patients’ survival and treatment response. However, it is important to emphasize that the association between the model presented herein and OS does not necessarily prove the role of these genes in combination in the pathogenesis of HER2-positive GC and these points require further studies. Nonetheless, results support the importance of the modulation of E-cadherin expression not only in the process of epithelial–mesenchymal transition (EMT) but also for metastases as recently underlined by Fand C. and Kan Y. [67], and indicated a relationship between E-CAD, HER2-overexpression, and patients’ survival. Data reported may be useful to better decipher the pathogenesis of HER2+ mGC and to direct drug research towards better therapies.

## Figures and Tables

**Table 1:** Clinicopathological characteristics of the patients with *HER2* positive/negative mGC (Series 1), patients with *HER2* negative GC metastatic/without metastasis (Series 2), patients generated by the TCAG Research network * correlating gene expression with patient’s overall survival (Series 3).

	Series 1	Series 2	Series 3
Characteristic	Number (%)	Number (%)	Number (%)
Patients	38	36	354
Country	Italy	Italy	USA
Etnia	Caucasic	Caucasic	White 224 (63.3%) Asian 73 (20.6%) Other 12 (3.4%) nv 45 (12.7%)
Median age (range)	69 (46–83)	61 (45–85)	67 (35–90)
Gender			
Male	25 (65.8%)	23 (63.9%)	229 (64.7%)
Female	13 (34.2%)	13 (36.1%)	125 (35.3%)
Lauren classification			
Intestinal	7 (18.4%)	8 (22.2%)	---
Diffuse	18 (47.4%)	23 (63.9%)	---
Adenocarcinoma-mix	13 (34.2%)	5 (13.9%)	---
GRADE			
Low	10 (26.3%)	10 (27.8%)	---
Moderate	10 (26.3%)	15 (41.7%)	---
High	10 (26.3%)	9 (25.0%)	---
Undifferentiate	8 (21.1%)	2 (5.6%)	---
Initial TNM stage			
Stage 1	1/34 (2.9%)	--	48 (14.2%)
Stage 2	3/34 (8.8%)	8 (22.2%)	110 (32.4%)
Stage 3	17/34(50.0%)	18 (50.0%)	146 (43.1%)
Stage 4	13/34 (38.2%)	10 (27.8%)	35 (10.3%)
missing	4	--	
GC with metastasis			
M0	--	26 (72.2%)	---
M1	38 (100.0%)	10 (27.8%)	---
Tumor location			
Upper	5 (13.2%)	7 (19.4%)	---
Middle	17 (44.7%)	17 (47.2%)	---
Lower	13 (34.2%)	9 (25.0%)	---
Whole-NAS	3 (7.9%)	3 (8.3%)	
HER2-positive #			
Yes	13 (34.2%)	--	71 (20.1%) °°°
No	25 (65.8%)	36 (100%)	283 (79.9%) °°°
Median Overall Survival ^$^			
at GC diagnosis	17 (95%CI 15.2–21.7)	36 (95%CI 16.0–83.0)	29 (95%CI 25.5–56.2)
at metastasis diagnosis	9 (95%CI 6.0–28.0)	14 (95%CI 7.0–91.1)	---

* https://www.cancer.gov/tcga, accessed on 3 May 2021; # IHC 3+ or IHC 2+/fluorescence or silver ISH+; °°° positive > 80 percentiles 37.61 FPKM; ^$^ survival in months. Nv = not evaluated.

**Table 2:** Nanostring analysis of gene mRNA expression level in GC series. The report displays gene differences in mRNA expression between HER2-positive and HER2-negative mGC (Series 1, *n* = 38), between metastatic and non-metastatic HER2-negative GC (Series 2, *n* = 36), and predictive power of gene value for OS generated by TCGA (Series 3, *n* = 354). Genes were listed by increasing the Series 1 *p*-value order (*t*-test).

	Series 1					Series 2		Series 3	
	HER2-Negative	HER2-Positive				mGC vs. GC			
	mRNA (SD)	mRNA (SD)	Difference	(95% CI)	*p*	Difference	*p*	*Best Cut-Off (FPKM)*	*p* ***
HER2	662.63 (267.54)	6494.43 (6611.22)	5831.80 (3180.43 to 8483.16)		0.0001	171.6005	0.3607	15.6	0.093
TP53	264.28 (100.17)	162.95 (82.44)	−101.33 (−166.95 to −35.71)		0.0034	248.4487	0.0280	26.26	0.008
RARA	191.88 (91.46)	363.31 (281.13)	171.43 (47.53 to 295.33)		0.0080	120.9811	0.3188	16.18	0.016
MLH1	279.30 (81.69)	207.04 (67.18)	−72.26 (−125.77 to −18.75)		0.0095	−11.0855	0.7260	5.08	0.044
EGF	4.25 (3.73)	19.21 (28.11)	14.95 (3.50 to 26.41)		0.0119	1.8393	0.4567	0.016	<0.0001
ZEB1	319.80 (232.65)	142.66 (93.25)	−177.14 (−314.06 to −40.22)		0.0127	71.8493	0.5166	6.15	0.0056
VCL	1168.13 (816.28)	611.60 (315.10)	−556.53 (−1035.64 to −77.42)		0.0240	−2.0464	0.9946	26.28	0.040
RXRA	488.90 (317.90)	271.43 (145.48)	−217.47 (−406.67 to −28.28)		0.0255	120.9811	0.3188	7.62	0.012
ATM	207.38 (120.23)	125.66 (53.69)	−81.72 (−153.11 to −10.33)		0.0260	−39.7789	0.5616	2.7	0.310
RPL19	17,988 (5299.35)	46,340.39 (62,340.74)	28,351.44 (3211 to 53,491)		0.0282	4555.8384	0.2742	338.18	0.131
WNT1	4.31 (2.52)	14.51 (22.32)	10.20 (1.15 to 19.25)		0.0282	0.6250	0.1977	0.11	0.017
KLRG1	24.78 (17.99)	12.29 (11.25)	−12.49 (−23.63 to −1.35)		0.0290	−11.8929	0.1798	0.86	0.021
MDM4	499.69 (266.15)	314.51 (176.44)	−185.18 (−351.62 to −18.74)		0.0302	45.8611	0.6586	3.45	0.120
SRC	1137.41 (882.62)	711.11 (335.50)	−426.30 (−816.04 to −36.55)		0.0330	590.5061	0.0439	17.08	0.023
SNAIL1	117.15 (55.26)	165.45 (84.07)	48.30 (2.35 to 94.26)		0.0399	112.8464	0.0009	3.8	0.004
SNAIL2	258.73 (182.49)	141.57 (131.76)	−117.16 (−233.18 to −1.14)		0.0479	56.8309	0.4574	4.85	0.002
MSH6	215.81 (44.97)	185.66 (39.37)	−30.14 (−60.093 to −0.19)		0.0486	*181.88*	0.035	3.2	0.120
WNT3	16.88 (9.55)	33.67 (40.78)	16.79 (−0.41 to 33.99)		0.0554	9.4464	0.0725	0.74	0.180
CTNND1	1920 (775.98)	1448.12 (537.48)	−471.79 (−961.05 to 17.46)		0.0583	711.6163	0.1020	50.2	0.072
B2M	49,959 (16,893)	63,541.36 (27,058.56)	13,581.78 (−870 to 28,034)		0.0647	10,086.9989	0.3130	684.8	0.241
CTNNB1	2638.01 (817.46)	2160.69 (664.70)	−477.32 (−1011 to 56)		0.0782	946.5439	0.0893	59.93	0.018
MMP2	2480.42 (1475)	1579.34 (1440.04)	−901.08 (−1916.38 to 114.22)		0.0803	393.2500	0.6960	23.97	0.016
WNT7a	8.75 (9.17)	19.83 (28.57)	11.080 (−1.48 to 23.64)		0.0821	−1.4464	0.6109	0.01	<0.0001
*CDH1*	1224.33 (615.21)	1618.05 (615.21)	393.71 (−70.07 to 857.50)		0.0937	683.5668	0.2511	91.73	0.120

FPKM: fragments per kilobase million; *TP53*, *SCR*, and *SNAIL1* were excluded from the next analysis since they were also found to be associated with HER2-negative cases according to the metastatic process (Series 2). *p* ***, Log-rank *p* value for Kaplan-Meier plot showing results from analysis of correlation between mRNA expression level and patient survival.

**Table 3:** Identification of mRNA expression genes associated with HER2-positive mGC in Series 1 (*n* = 13) discriminating patients with lower survival and higher survival. The median time from diagnosis of GC in HE2-positive mGC was 22 months. ROC curve analysis identified *EGF, CTNND1, SRC, TP53, SNAI1, RPL19*, and *RARA* as genes that discriminated patients based on OS from diagnosis of GC (AUC > 0.800). Kaplan–Meier univariate analysis and stepwise Cox multivariate analysis were performed. A *p*-value of less than 0.05 was used as a cutoff to define and select the patients’ OS-related genes in the *Wnt/β-catenin* pathway. *RARA* and *CTNND1* resulted as two independent factors by multivariate analysis to predict OS in HER2-positive mGC at the time of GC diagnosis.

					Univariate Analysis		Multivariate Analysis	
Variable	AUC	SE	95% CI	Best Cut-Off	HR (95% CI)	*p*-Value	HR (95% CI)	*p*-Value
HER2	0.905	0.0999	0.616 to 0.996					
EGF	0.929	0.0696	0.647 to 0.999	>2.51	0.053 (0.008 to 0.334)	0.002		--
CTNND1	0.881	0.105	0.587 to 0.991	>1470.73	0.074(0.014 to 0.377)	0.002	0.0217 (0.001 to 0.352)	0.007
SRC **	0.857	0.113	0.558 to 0.984					--
TP53 **	0.857	0.123	0.558 to 0.984					--
SNAIL1 **	0.833	0.128	0.531 to 0.976					--
RPL19	0.833	0.124	0.531 to 0.976	>17,216	0.111(0.028 to 0.447)	0.002		--
RARA	0.81	0.137	0.505 to 0.967	>260.21	0.173(0.044 to 0.68)	0.012	0.0782 (0.009 to 0.68)	0.0211
RXRA	0.738	0.152	0.430 to 0.934					
EGFR	0.69	0.162	0.384 to 0.908					
SNAIL2	0.667	0.162	0.362 to 0.894					
KLRG1	0.643	0.18	0.340 to 0.879					
ZEB1	0.643	0.177	0.340 to 0.879					
GSK3B	0.643	0.184	0.340 to 0.879					
*CDH1*	0.619	0.173	0.319 to 0.864					
B2M	0.595	0.172	0.298 to 0.848					
WNT7A	0.595	0.173	0.298 to 0.848					
ATM	0.595	0.181	0.298 to 0.848					
CTNNB1	0.571	0.182	0.278 to 0.831					
VCL	0.571	0.183	0.278 to 0.831					
WNT1	0.571	0.191	0.278 to 0.831					
WNT3	0.56	0.206	0.268 to 0.823					
MSH6	0.548	0.183	0.259 to 0.814					
MLH1	0.548	0.176	0.259 to 0.814					
MMP2	0.5	0.178	0.221 to 0.779					
MDM4	0.5	0.224	0.221 to 0.779					

AUC, the area under the curve; SE, standard error; HR, hazard ratio; CI, confidence interval; OS, overall survival. ** *SRC, TP53*, and *SNAI1* were excluded from the analysis since they were found associated with metastasis in the HER2-negative subtype and thus considered not specific to HER2-overexpression.

**Table 4:** After the diagnosis of metastasis, *RARA, EGF*, and *RPL19* expression discriminated between low-survival and high-survival groups (ROC curve analysis, AUC > 0.700), but *RARA* expression did not reach a statistical significance in univariate analysis. Multivariate Cox regression model revealed *EGF* as an independent factor of OS after the diagnosis of metastases.

					Univariate Analysis		Multivariate Analysis	
Variable	AUC	SE	95% CI	Best Cut-Off	HR (95% CI)	*p*-Value	HR (95% CI)	*p*-Value
HER2	0.81	0.165	0.505 to 0.967					
RARA	0.801	0.083	0.640 to 0.912	>285.16	0.426 (0.131 to 1.371)	ns		
EGF	0.747	0.101	0.580 to 0.874	>3.83	0.177 (0.041 to 0.775)	0.0207	0.220 (0.0053 to 0.9158)	0.037
RPL19	0.716	0.111	0.547 to 0.850	>22,791	0.212 (0.0561 to 0.780)	0.0220		
TP53	0.682	0.121	0.511 to 0.823					
MLH1	0.643	0.168	0.340 to 0.879					
SRC	0.621	0.103	0.449 to 0.773					
KLRG1	0.619	0.173	0.319 to 0.864					
ZEB1	0.595	0.181	0.298 to 0.848					
WNT3	0.595	0.181	0.298 to 0.848					
B2M	0.595	0.176	0.298 to 0.848					
MSH6	0.595	0.176	0.298 to 0.848					
CTNND1	0.59	0.096	0.419 to 0.747					
WNT1	0.571	0.191	0.278 to 0.831					
MMP2	0.571	0.175	0.278 to 0.831					
*CDH1*	0.571	0.191	0.278 to 0.831					
MDM4	0.569	0.127	0.399 to 0.728					
MDM2	0.548	0.121	0.378 to 0.710					
VCL	0.548	0.179	0.259 to 0.814					
RXRA	0.548	0.187	0.259 to 0.814					
SNAIL2	0.548	0.209	0.259 to 0.814					
CTNNB1	0.548	0.176	0.259 to 0.814					
EGFR	0.536	0.112	0.368 to 0.699					
ATM	0.524	0.179	0.240 to 0.797					
GSK3B	0.524	0.187	0.240 to 0.797					
SNAI1	0.519	0.122	0.352 to 0.684					
WNT7A	0.5	0.190	0.221 to 0.779					

AUC, the area under the curve, SE, standard error; HR, hazard ratio; CI, confidence interval; OS, overall survival; ns, no significance.

**Table 5:** Univariate and stepwise multivariate Cox regression analyses to validate HER2-related genes associated with OS rate in TCGA data set.

	Univariate Analysis			Multivariate Analysis		
Covariate	b	SE	*p*	b	SE	*p*
RPL19	0.0003035	0.0002348	0.1962			
RARA	−0.0004683	0.004318	0.9136			
KLRG1	0.05449	0.6961	0.9376			
WNT1	0.05449	0.6961	0.9376			
ZEB1	0.0508	0.0529	0.3369			
SNAI2	0.06641	0.08788	0.4498			
EGF	0.3819	0.1358	0.0049	0.4320	0.1454	0.0030

## Data Availability

The Human Protein Atlas (TCGA) dataset is an open public resource https://www.proteinatlas.org/, accessed on 3 May 2021 and licensed under the Creative Commons Attribution-ShareAlike 3.0 International License. Details regarding data supporting reported results were generated during the study and listed in the manuscript.

## Supplementary Materials

The following supporting information can be downloaded at: https://www.mdpi.com/article/10.3390/cancers14051266/s1, Figure S1: Comparison of *HER2* and *CDH1* mRNA expression levels with HER2-positivity; Figure S2: Distribution of serum sE-CAD in blood donors (BD, n = 44) and in patients with GC (n = 94) and according to the rs16260 variants. Figure S3: The Median expression of *CDH1* mRNA level increased in patients having simultaneously the rs16260-A variant and high E-CAD expression in immunohistochemistry, and HER2-negative mGC, patients having the wild type *CDH1* (rs16260 C/C) showed a reduced survival compared to patients with HER2-positive mGC. Figure S4: String protein-protein interaction network covering genes we found specifically associated with mRNA HER2-overexpressed GC.

## Appendix A

**Table A1:** Forthy four genes included in the Nanostring mRNA panel.

*ATM*	*KLRG1*	*RARA*	*TNFAIP8L3*	*B2M*
*CDH1*	*MDM2*	*RARB*	*TP53*	*GAPDH*
*CDKN1A*	*MDM4*	*RARG*	*TWIST1*	*HPRT1*
*CTNNB1*	*MLH1*	*RXRA*	*VCL*	*RPL19*
*CTN* *ND1*	*MMP2*	*RXRB*	*WNT1*	
*EBER1*	*MMP9*	*RXRG*	*WNT2*	
*EGF*	*MSH2*	*SNAI1*	*WNT3*	
*EGFR*	*MSH6*	*SNAI2*	*WNT5a*	
*GSK3B*	*PIK3CA*	*SRC*	*WNT7a*	
*HER2*	*PMS2*	*TNFAIP8L1*	*ZEB1*	
