# Supplementary material for: HER2–CDH1 Interaction via Wnt/B-Catenin Is Associated with Patients’ Survival in HER2-Positive Metastatic Gastric Adenocarcinoma
Source: Cancers (Basel). 2022 Feb 28;14(5):1266. doi: 10.3390/cancers14051266 (PMC8909509; doi:10.3390/cancers14051266)

cancers

MDPI

Supplementary figures

Figure S1. Comparison of HER2 and CDH1 mRNA expression levels with HER2-positivity **(A)** and E-CAD immunohistochemical evaluation **(B)** in the same tumor cases. The median *HER2* mRNA expression level was strongly increased in the HER-POS group, Kruskal-Wallis, P<0.001. The median *CDH1* mRNA expression level increased in patients with E-cadherin score 2 compared with those having a reduced E-cadherin expression (score 1) and much more with those having a low E-cadherin expression (score 0) at immunohistochemical staining. Kruskal-Wallis, P=0.039, Jonckheere trend test, P=0.035.

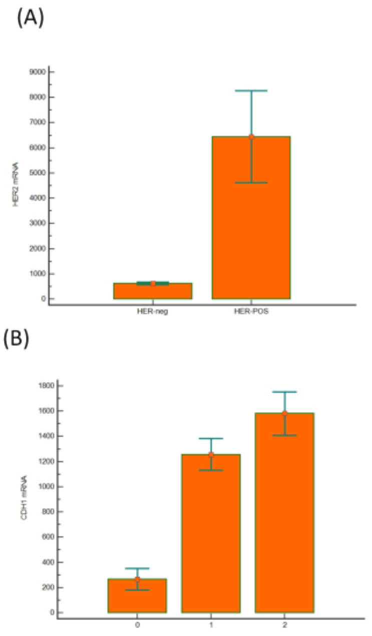

Figure S2. a) Distribution of serum sE-CAD in blood donors (BD, n=44) and in patients with GC (n=94). The median serum levels of sE-CAD were elevated in both HER2-neg mGC *(4885 pg/ml) and HER2-Pos mGC (6001 pg/ml)*, and they were similar between BD *(4187pg/ml)* and GC without metastasis *(3033 pg/ml)*. After treatment, t*he* concentration of sE-CAD decreased in both the m GC groups [Difference prior and after treatment: HER2-negative: GC m I *vs* GC m II: -2541, (95%CI -3567 to -1100); HER2–positive: GC-H m I *vs* GC-H m II: -3667 (95%CI -5791 to -639)].

sCDH1, soluble E-cadherin form; GC I, GC without metastasis (n=49), GC m I, HER2-negative metastatic GC before treatment (n=49) GC-H-m I HER2-positive metastatic GC before treatment (n=6), GC-H m II, HER2-negative metastatic GC after 3 weeks of the initial treatment (n=42); GC-H m II, HER2-positive metastatic GC after 3 weeks of the initial treatment (n=5). *, p<0.05.

b) Distribution of serum sE-CAD in the same groups of patients according to the rs16260 variants.

a)

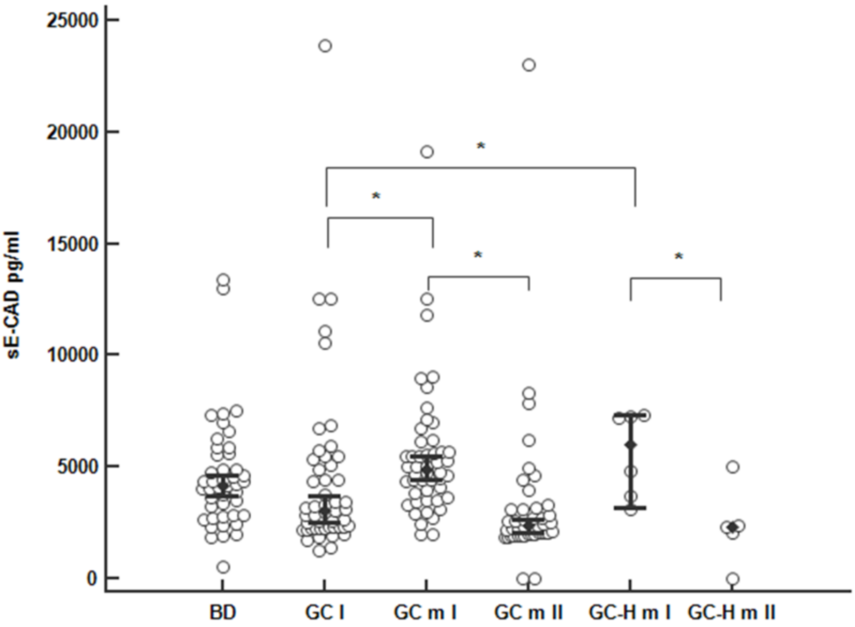
25000
20000
15000
10000
5000
0
sE-CAD pg/ml
*
*
*
*
BD
GC I
GC m I
GC m II
GC-H m I
GC-H m II

b)

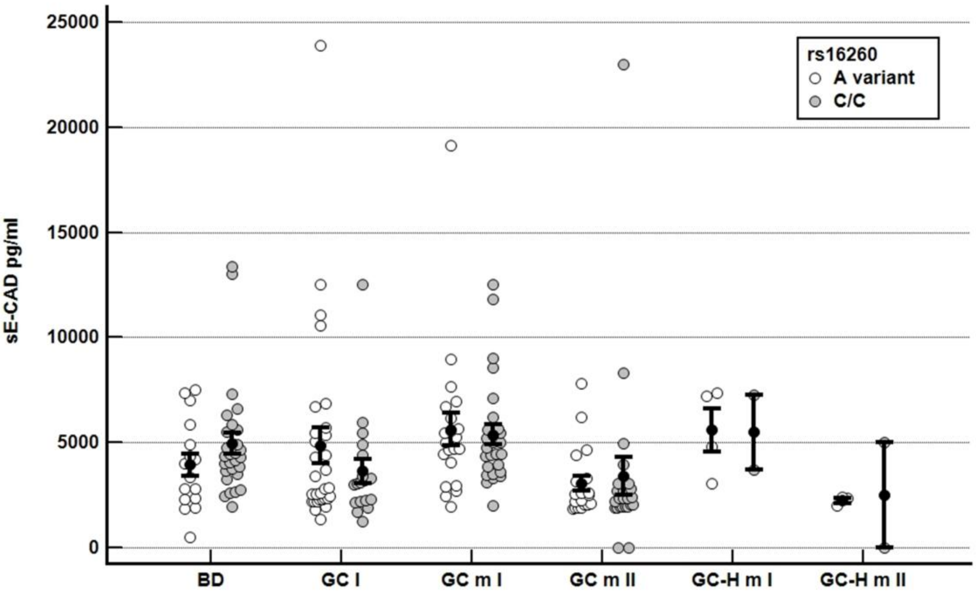
25000
20000
15000
10000
5000
0
sE-CAD pg/ml
rs16260
A variant
C/C
BD
GC I
GC m I
GC m II
GC-H m I
GC-H m II

Figure S3. The Median expression of *CDH1* mRNA level increased in patients having simultaneously the rs16260-A variant (A/A + A/C genotype) and high E-CAD expression (IHC staining score 2) (**A,** P<0.05). In the same way, the median expression of HER2 mRNA level increased in patients having simultaneously the rs16260-A variant (A/A + A/C genotype) and high HER2 expression (HER2-POS) (**C,** P<0.05). Neither the CDH1 mRNA expression levels nor the HER2 mRNA reached any statistical difference in change concerning *CDH1* rs1801552 genotypes (**B, D**) (Two way Anova tests). the number of patients with rs16260 A variant was higher in the HER2-positive than in HER2-negative mGC patients (11 of 24, 45.8% and 7 of 24, 29.1%, respectively). The number of patients with rs16260 A variant was higher in the HER2-positive than in HER2-negative mGC patients (11 of 24, 45.8% and 7 of 24, 29.1%, respectively) **(E)**. HER2-negative mGC, patients having the wild type CDH1 (rs16260 C/C) showed a reduced survival compared to patients with HER2-positive (**F**).

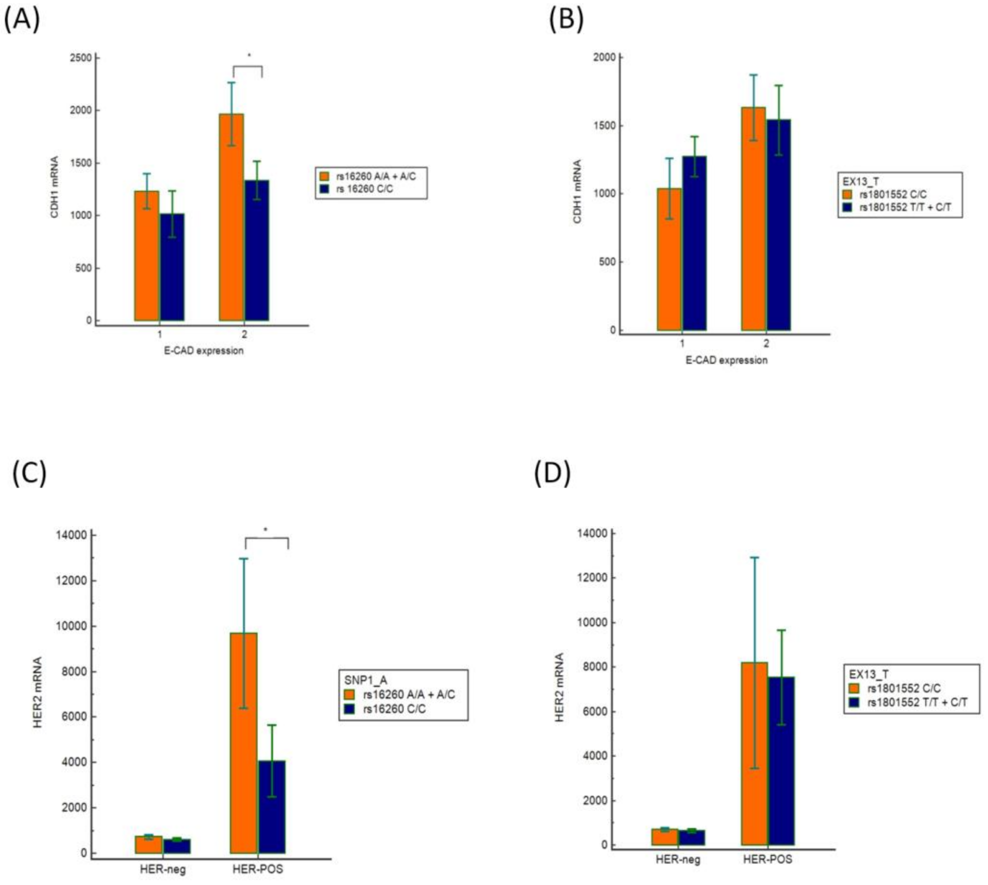

e)

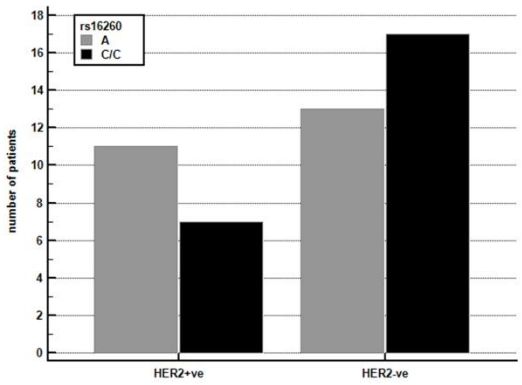

f)

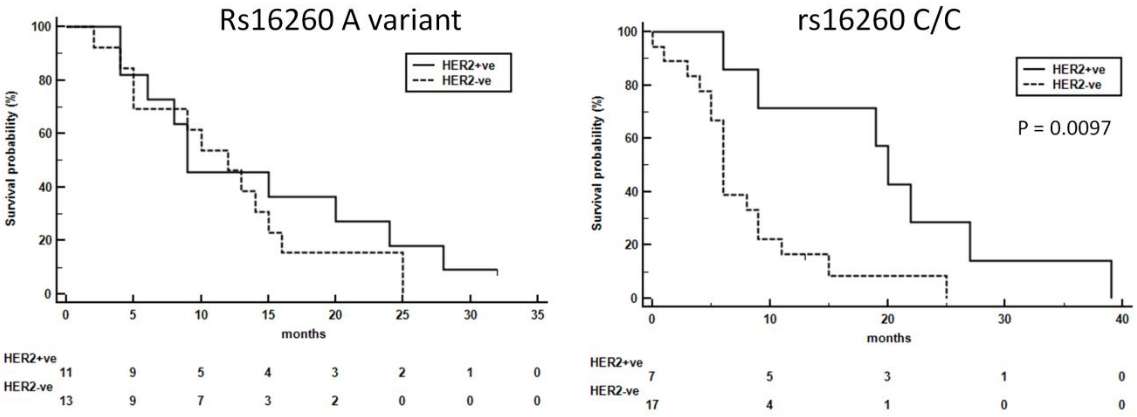

Figure S4. String protein-protein interaction network covering genes we found specifically associated with mRNA HER2-overexpressed GC (i.e. *RPL19, KLRG1, RARA, ZEB1, SNAI2, CDH1,* and *EGF)*

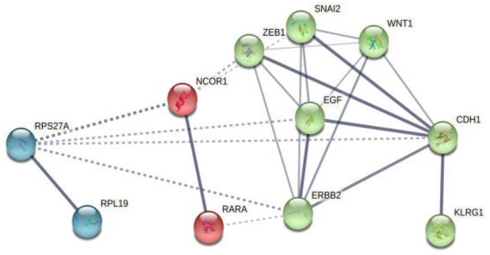

Supplement: Supplementary file 1 [file cancers-14-01266-s001.zip › cancers-1552460-supplementary.pdf]
